# Effect of ketogenic diet on exercise tolerance and transcriptome of gastrocnemius in mice

**DOI:** 10.1515/biol-2022-0570

**Published:** 2023-02-23

**Authors:** Jie Zhang, Bo Chen, Ke Zou

**Affiliations:** Department of Police Physical Training, Zhejiang Police Collage, Zhejiang, China; Department of Physical Education, Beijing University of Chemical Technology, 15 North Third Ring East Road, Chaoyang District, Beijing, 100029, China; School of Physical Education, Huaibei Normal University, Anhui, China

**Keywords:** ketogenic diet, exercise tolerance, lipid metabolism, high-throughput sequencing

## Abstract

Ketogenic diet (KD) has been proven to be an optional avenue in weight control. However, the impacts of KD on muscle strength and exercise endurance remain unclear. In this study, mice were randomly allocated to normal diet and KD groups to assess their exercise tolerance and transcriptomic changes of the gastrocnemius. KD suppressed body-weight and glucose levels and augmented blood ketone levels of mice. The total cholesterol, free fatty acids, and β-hydroxybutyric acid levels were higher and triglycerides and aspartate aminotransferase levels were lower in KD group. There was no notable difference in running distance/time and weight-bearing swimming time between the two groups. Furthermore, KD alleviated the protein levels of PGC-1α, p62, TnI FS, p-AMPKα, and p-Smad3, while advancing the LC3 II and TnI SS protein levels in the gastrocnemius tissues. RNA-sequencing found that 387 differentially expressed genes were filtered, and Cpt1b, Acadl, Eci2, Mlycd, Pdk4, Ptprc, C1qa, Emr1, Fcgr3, and Ctss were considered to be the hub genes. Our findings suggest that KD effectively reduced body weight but did not affect skeletal muscle strength and exercise endurance via AMPK/PGC-1α, Smad3, and p62/LC3 signaling pathways and these hub genes could be potential targets for muscle function in KD-treated mice.

## 1 Introduction

The classic definition of the ketogenic diet (KD) is a formula diet with high fat, low carbohydrate, and normal protein [1]. Compared with an ordinary diet, the body mainly relies on the ketone body produced by fat oxidation for energy after consuming KD [2]. The ketone body was composed of 78% β-hydroxybutyrate (β-HB), 20% acetoacetic acid, and 2% acetone [3]. The ketone body is a normal intermediate product of fatty acid decomposition and oxidation in the liver, its molecule is small and easily soluble in water, and can pass through the capillary wall of muscle and the blood–brain barrier, which is an important source of energy in muscle, especially brain tissues [4]. In case of long-term starvation or insufficient sugar supply, the ketone body could not only replace glucose (Glu) and become a significant energy source for metabolic tissues such as the brain and muscle but also participate in the modulation of various metabolic pathways of the body as signal molecules [5,6].

The classic KD was first put forth in 1921 [1]. Except for classic KD, there were numerous types of KDs such as using medium chain triglycerides, long chain triglycerides, and low glycemic index treatment [1]. With the ongoing advancement of research, a previous report clarified that KD has been developed and successfully taken to treat epilepsy, especially to reduce seizures in children who do not respond to pharmacological interventions [7]. On the other side, Marchiò et al. found that ghrelin and des-acyl ghrelin were downregulated in children affected by refractory epilepsy treated with the KD in a short term or even after 1 year, which may associate with growth retardation in children [8,9]. In addition, scientific research confirmed that KD relieved the hyperglycemia symptoms of type 2 diabetes mellitus by producing ketone bodies and using ketone bodies for energy [10]. Growing evidence has extended KD to metabolic diseases such as obesity, fatty liver, cardiovascular, and genetic kidney diseases, but also certain types of cancer [11–13]. It is widely known that this dietary method reduces body weight, the most famous of which is the “Atkins diet,” whose effectiveness in weight control has been confirmed [14,15]. High-carbohydrate (CHO) diets have long been used as a common dietary strategy to ensure that athletes have adequate muscle glycogen stores for better performance during training or pre-competition. However, the use of high-CHO dietary strategies exposes athletes to a higher risk of chronic diseases such as diabetes and cardiovascular disease. Research indicated that KD for a while contributes to converting the way the body uses energy, from carbohydrate-centered to fat-based energy production, i.e., “fat adaptation” [16,17]. KD could efficiently lower body weight, lower fasting blood Glu, minimize oxidative stress brought on by exercise, and promote adipose tissue oxidation when compared to a high-CHO diet [15–17]. Additionally, recent research has demonstrated that KD has neuroprotective effects, ameliorates mitochondrial content and function, activates autophagy, and enhances antioxidant and anti-inflammatory effects [18–22]. However, as a special dietary pattern, whether KD affects skeletal muscle strength and exercise endurance has not been reported.

As we know, KD has a better treatment effect for a variety of diseases, including tumors, diabetes, and asthma. The mechanism of action of KD is complex. For tumors, tumor growth can be inhibited by enhancing oxidative stress, cellular autophagy, and reducing blood Glu and glucose transporter 1 expression levels [22]. In terms of neurological diseases, the protective effect of KD was associated with inhibiting the inflammatory response, enhancing mitochondrial function and improving oxidative stress. Generally, KD has important clinical applications as an adjunctive dietary treatment method. Although numerous clinical studies indicate the potential and advantages of KD in patients or athletes. Nevertheless, KD the participating molecules and events that determine the exercise endurance in athletic sports have not yet been fully elucidated.

In this study, C57BL/6 mice were fed KD and a normal diet (ND) for 4 weeks, respectively, to observe and measure the exercise endurance and lipid metabolism level of mice. Meanwhile, transcriptome sequencing was used to analyze the effect of KD on genomic changes in gastrocnemius of C57BL/6 mice. The present study aimed to determine the effect and potential mechanism on strength and exercise endurance of skeletal muscle in mice treated with a KD.

## 2 Materials and methods

### 2.1 Animals

Twenty male C57BL/6 mice with SPF grade (6–8-week old, weight varies from 18 to 22 g) were introduced from Shanghai SLAC Laboratory Animal Co., Ltd (SCXK (Hu) 2013-0016, Shanghai, China). All mice were reared in an SPF laboratory animal barrier environment with temperature (20–25°C), humidity (40–60%), and light (12 h light/dark cycle).


**Ethical approval:** The research related to animal use has been complied with all the relevant national regulations and institutional policies for the care and use of animals.

### 2.2 Experimental design

After 1 week of adaptive feeding, mice were randomly divided into the ND and KD groups, with ten mice in each group. Among them, ketogenic feed was purchased from Beijing Botai Hongda Biological Co., Ltd (D12358, Beijing, China) and standard feed was acquired from the Experimental Animal Center of Xi’an Jiaotong University (D12450B, Xian, China). Each animal was fed in a single cage and their body weight and food intake were recorded daily. Animals in the ND group were given a conventional diet *ad libitum*, whereas those in the KD group received a KD of equivalent energy based on the average of food consumption in the first 24 h in the ND group for a period of 4 weeks. All animals utilized in this experiment were allowed by the Ethics Committee of Hangzhou Eyong Biotechnological Co., Ltd Animal Experimental Center (SYXK (Zhe) 2021-0033). We spared no effort to mitigate the suffering of animals during the experiments.

### 2.3 Determination of blood Glu and blood ketone

After 4 weeks of KD, we harvested blood samples from the tail vein of mice. Glu and blood ketone were determined via a blood ketone meter (Abbott Diabetes Care, USA) and blood glucose meter (580, Yuwell, China), respectively.

### 2.4 Assessment of blood biochemical indexes

After 4 weeks of KD, the harvested blood samples underwent centrifugation (5,000 rpm, 20 min). The obtained supernatant was known as serum. Serum total cholesterol (TC) kit (CS0005), triglycerides (TG) kit (TR0100), free fatty acids (FFA) kit (MAK044), β-HB acid kit (MAK041), aspartate aminotransferase (AST) kit (MAK055), and alanine aminotransferase (ALT) kit (MAK052) were introduced from Sigma-Aldrich (USA). All experimental operations were done in strict accordance with the instructions of the corresponding kit to evaluate the content of blood biochemical indexes.

### 2.5 Endurance test

Seven days before exhaustive exercise, each mouse ran on a treadmill for 10 min at a speed of 15 m/min. The endurance test was carried out on an open treadmill (Natsume, Kyoto, Japan). The endurance test started at 10 m/min for 15 min, followed by 15 and 20 m/min for 15 min each, and then 24 m/min and 7% grade till exhaustion. We recorded the running distance and running time.

### 2.6 Weight-bearing swimming experiment

After 4 weeks of KD, mice were subjected to a weight-bearing swimming test to assess the endurance of mice [23]. The homogeneous iron ring was placed 2 cm away from the tail tip of the mouse, and the effective weight bearing was 3% of the body weight. The water temperature and water depths were 25 ± 1°C and 50 cm, respectively. Depletion time was determined by recording each mouse from the time point at which it was unable to surface within 10 s of release to submersion. Furthermore, the levels of Glu and blood ketone in mice were detected before and within 3 min after weight-bearing swimming.

### 2.7 Hematoxylin–eosin (H&E) staining

After the weight-loaded forced swimming test, each mouse was euthanized by inhaling excess CO_2_. The gastrocnemius was quickly removed. The gastrocnemius tissue fixation was done with the aid of 10% formalin (E672001, Sangon, China). Following dehydration and embedded in paraffin, the tissues were sectioned to 5 µm thick. After that, the slices were dewaxed and hydrated. After being stained with hematoxylin (BL700A, Biosharp, China) for 5 min, differentiation was performed with 1% hydrochloric acid for 30 s. After counterstaining with eosin for 3 min, the slices were dehydrated. We chose xylene to permeate the slices. Pathological changes of gastrocnemius in mice were monitored with the help of an optical microscope (ECLIPSE 80i, Nikon, Japan).

### 2.8 Western blotting

The acquired gastrocnemius tissues were lysed with the assistance of a lysis buffer (abs9229, absin, China). Subsequently, we selected the BCA kit (BI-WB005, SBJBIO, China) to quantify the lysed protein. Thereafter, the quantified protein was electrophoresed for protein separation, which was then loaded onto the PVDF membrane (PW0034, Leagene, China). After being sealed in 5% bovine serum albumin (BL-082, SBJBIO, China) at 37°C for 60 min, the membrane underwent primary antibodies (4°C, overnight). Afterward, the bound antibodies were then exposed to anti-rabbit secondary antibody (31466, Invitrogen, USA) or anti-mouse secondary antibody (S0002, Affinity, USA) with the condition of 37°C for 60 min. Visualization of protein was conducted by applying an ECL reagent (GK10008, GlpBio, USA) on a gel imaging system (A44114, Invitrogen, USA). The primary antibodies of PGC-1α (1:1,000, ab191838), P62 (1:10,000, ab109012), LC3B (1:2,000, ab48394), AMPKα (1:5,000, ab32047), p-AMPKα (1:10,000, ab133448), Smad3 (1:1,000, ab208182), p-Smad3 (1:2,000, ab52903), and β-actin (1:5,000, ab8227) were provided by Abcam (UK).

### 2.9 RNA extraction and library construction for RNA-seq

To check the role of KD on the genomic changes of mice, an RNA-seq assay was done on gastrocnemius tissues of ND mice and KD mice. Total RNA extraction was conducted with Trizol (10296010, Invitrogen, USA). For the detection of the quality and purity of total RNA, we applied NanoDrop 2000 (Thermo Scientific, USA). The integrity of RNA was measured by using Bioanalyzer 2100 (Agilent, CA, USA). Next, sequencing libraries were conducted by applying TruSeq RNA Library Prep Kit v2 (RS-122-2001/2, Illumina, USA). Afterward, the library was paired-end sequenced in a Hiseq 2500 platform (Illumina, USA).

### 2.10 Raw data filtering

The sequencing data contained some reads with adapters and low quality, which would cause a great interference to the subsequent information analysis. Therefore, the sequencing data should be further filtered. The criteria for data filtering majorly included the application of Cutadapt to remove sequences with adapters at the 3′ end and reads with an average quality score below Q20.

### 2.11 Differential expression analysis

We adopted R package DESeq2 to analyze significant differences in gastrocnemius samples between ND group and KD group. The conditions for screening differentially expressed genes (DEGs) were expression difference fold |log 2^FoldChange^| >1 and significant *P*-value <0.05.

### 2.12 Clustering analysis

The Pheatmap software package in R language was applied for bidirectional clustering analysis of DEGs in ND and KD samples. Cluster analysis was conducted based on the expression level of the same gene in different specimens and the expression pattern of different genes in the same specimen. The Euclidean method was taken to calculate the distance, and the hierarchical clustering longest distance method was exploited for clustering.

### 2.13 Functional enrichment analysis and construction of protein–protein interaction (PPI) network

For identification of mRNAs in the ND group and KD group, Gene Ontology (GO) and Kyoto Encyclopedia of Genes and Genomes (KEGG) enrichment analyses were done by applying Cytoscape plug-in ClueGO (http://apps.cytoscape.org/apps/cluego). After that, the STRING database (https://string-db.org/) was selected to analyze the protein interaction of the predicted target genes. The MCODE plug-in in Cytoscape was exploited to construct a PPI network.

### 2.14 Statistical analysis

**Figure 1:**
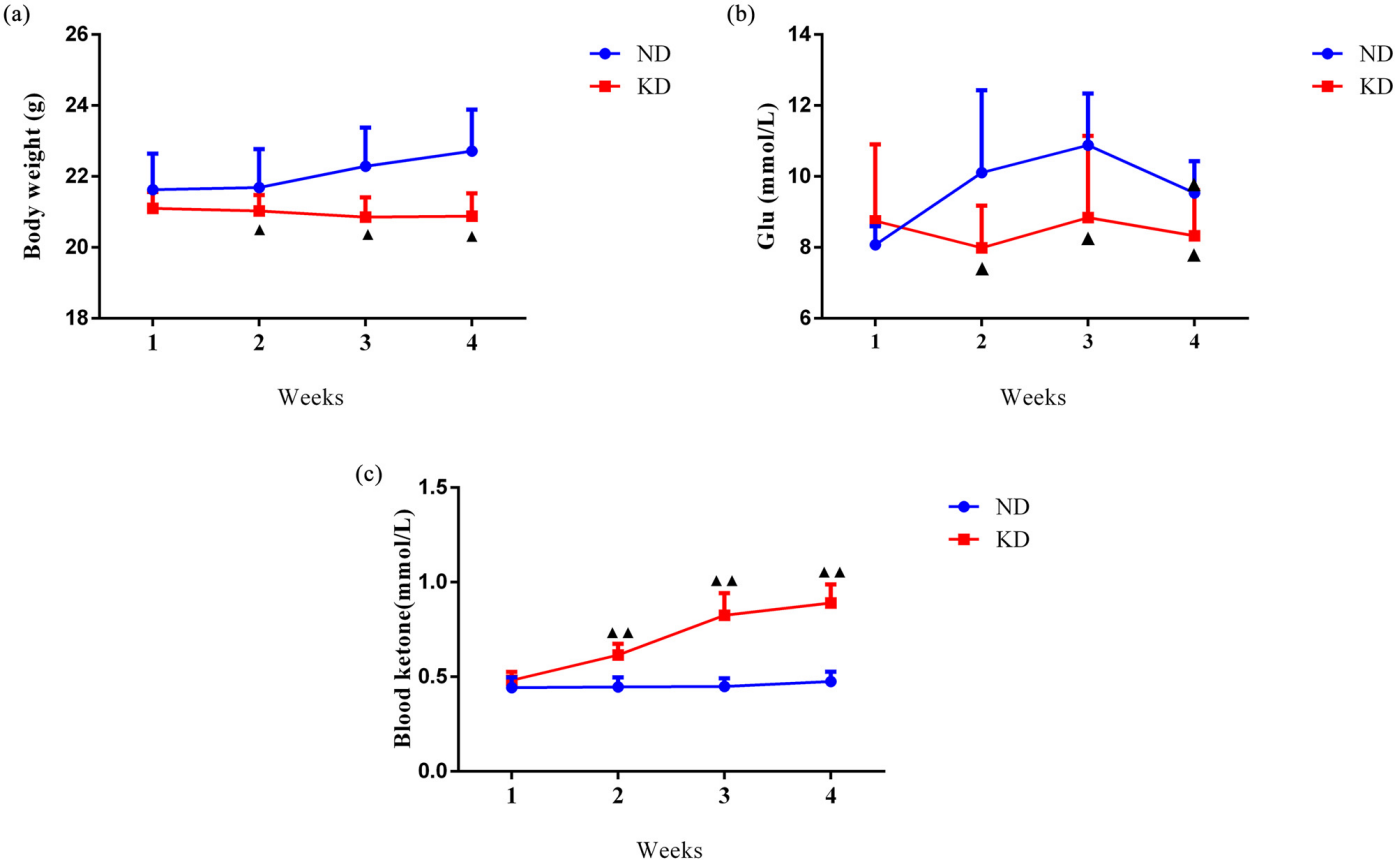
KD suppressed body weight and Glu level and augmented blood ketone level of C57BL/6 mice. (a) Body weight trajectories of mice on KD for 4 weeks. (b) Blood Glu level of mice on the KD for 4 weeks. (c) Blood ketone level of mice on the KD for 4 weeks. Data are expressed as mean ± SD (*n* = 10/group). Statistical significance was determined by one-way ANOVA. ^▲^ *P* < 0.05, ^▲▲^ *P* < 0.01 vs ND group. ND, normal diet; KD, ketogenic diet.

All quantitative data were described as mean ± standard deviation (SD). Data comparisons were done with the aid of a one-way analysis of variance for multiple time points with Tukey’s *post hoc* test in Figure 1 by using SPSS 16.0 (IBM, SPSS, USA). Kruskal–Wallis *H* test was applied to the heterogeneity of variance. Statistical analysis between the two groups was tested by a two-tailed Student’s *t*-test. Statistically, *P* < 0.05 was meaningful.

## 3 Results

### 3.1 KD suppressed body weight and Glu level and augmented blood ketone level of C57BL/6 mice

The results of body weight monitoring clarified that from the second week, the body weight of mice in the KD group was lower relative to the ND group (Figure 1a, *P* < 0.05). We also discovered that KD led to a decrease in Glu level from the second week (Figure 1b, *P* < 0.05). Meanwhile, compared to the ND group, the blood ketone level in the KD group was largely upregulated (Figure 1c, *P* < 0.01).

### 3.2 Effect of KD on lipid metabolism levels in C57BL/6 mice

**Figure 2:**
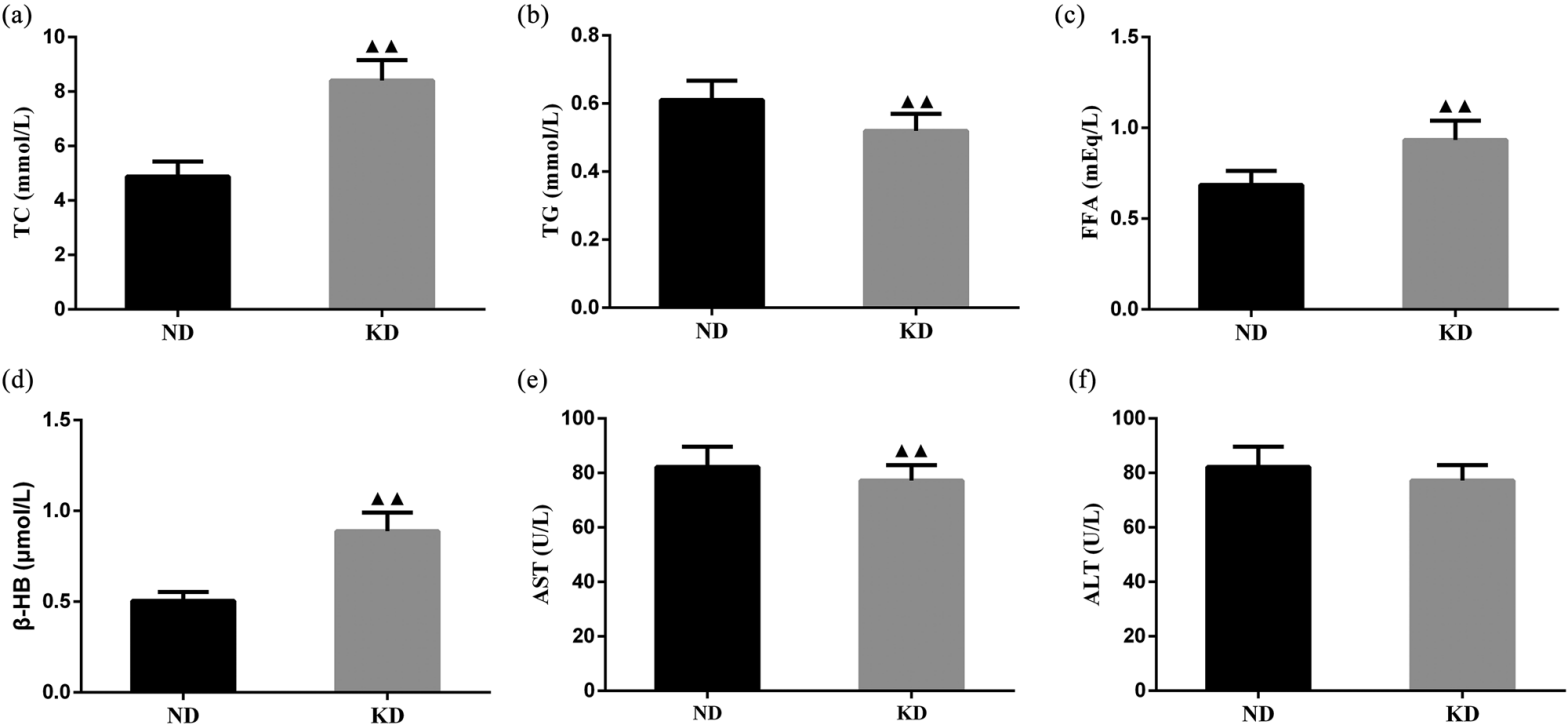
Effect of KD on lipid metabolism levels in C57BL/6 mice. Levels of (a) TC, (b) TG, (c) FFA, (d) β-HB, (e) AST, and (f) ALT in serum samples of mice treated with KD for 4 weeks. Data are expressed as mean ± SD. Differences of data in mice were assessed by two-tailed Student’s *t*-test. ^▲▲^ *P* < 0.01 vs ND group. ND, normal diet; KD, ketogenic diet.

By conducting an ELISA assay, we found that relative to the ND group, the results elucidated that the contents of serum TC, FFA, and β-HB were higher and the contents of serum TG and AST were lower in the KD group (Figure 2a–e, *P* < 0.01). There was no obvious difference in serum ALT levels between the KD group and the ND group (Figure 2f).

### 3.3 Effect of KD on exercise tolerance in C57BL/6 mice

**Figure 3:**
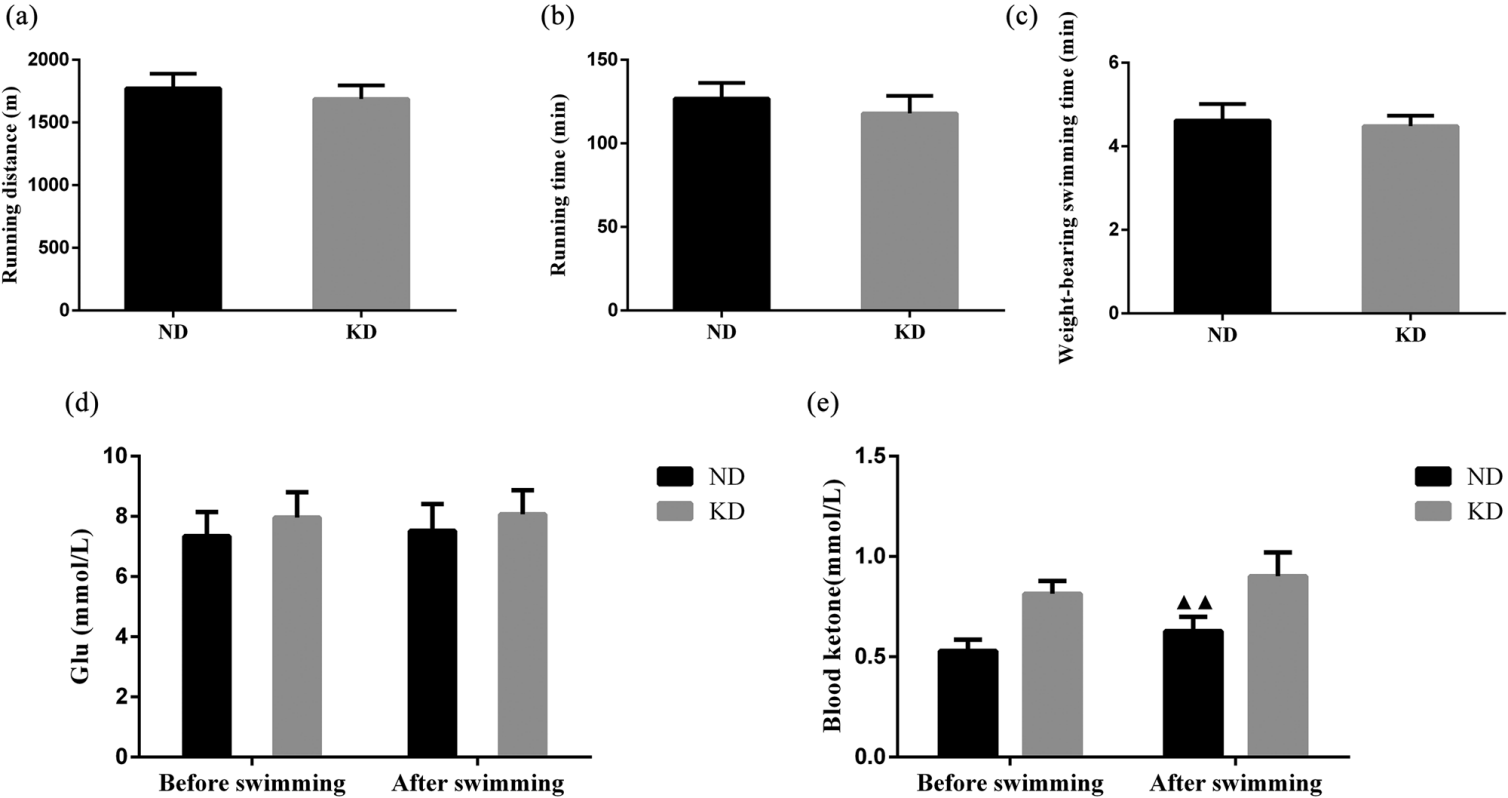
Effect of KD on exercise tolerance in C57BL/6 mice. (a) Running distance, (b) running time, (c) weight-bearing swimming time of mice treated with KD for 4 weeks. (d) Levels of blood Glu and (e) ketone in mice before and after swimming. Data are expressed as mean ± SD. Differences of data in mice were assessed by two-tailed Student’s *t*-test. ^▲▲^ *P* < 0.01 vs ND group. ND, normal diet; KD, ketogenic diet.

The endurance test results revealed that there was no notable difference in running distance and running time between KD group and ND group (Figure 3a and b). Also, a weight-loaded forced swimming test result unveiled that the weight-bearing swimming time of the KD group and ND group mice had no prominent change (Figure 3c). Furthermore, there was no evident change in Glu level before and after swimming in the KD group and ND group (Figure 3d). The blood ketone level of mice in the ND group after weight-bearing swimming was higher than that before swimming (Figure 3e, *P* < 0.01), while there was no notable change in blood ketone level in the KD group before and after weight-bearing swimming.

### 3.4 Effect of KD on pathological changes, muscle fiber-, and autophagy-related proteins of gastrocnemius tissues in mice

**Figure A1:**
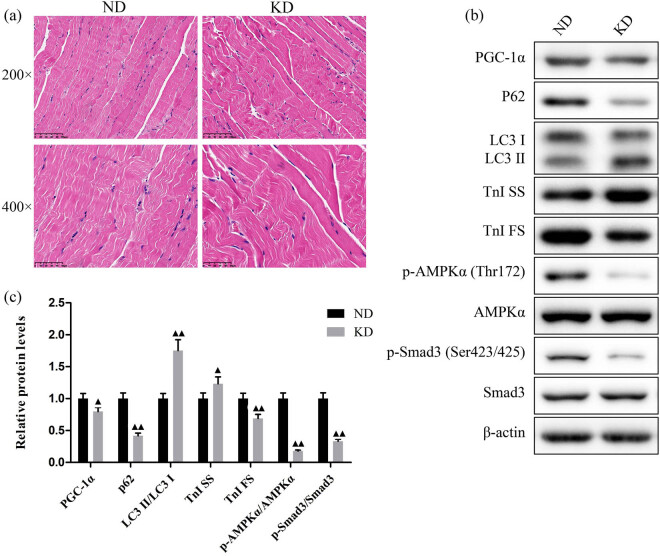
Effect of KD on pathological changes, muscle fiber, and autophagy-related proteins of gastrocnemius tissues in mice. (a) HE staining of the gastrocnemius tissues showed that KD had no differential influence in mice. (b) Representative protein bands and (c) quantification data of PGC-1α, p62, LC3, TnI SS, TnI FS, p-AMPKα, AMPKα, p-Smad3, and Smad3 in gastrocnemius tissues of mice treated with KD for 4 weeks. Data are expressed as mean ± SD. Differences of data in mice were assessed by two-tailed Student’s *t*-test. ^▲^ *P* < 0.05, ^▲▲^ *P* < 0.01 vs ND group. ND, normal diet; KD, ketogenic diet.

In this part, we monitored the pathological changes of gastrocnemius tissues in the KD group and ND group by H&E staining. Our results demonstrated that there were no obvious pathological changes in the gastrocnemius of mice in the KD and ND groups (Figure A1a). To further explore the relationship between exercise endurance and muscle fiber type, the autophagy, AMPK/PGC-1α, and Smad3 pathway-associated markers were measured. Western blot experiment documented that KD was found to alleviate the protein levels of PPARγ coactivator-1α (PGC-1α), sequestosome 1 (p62/SQSTM1), and troponin I-FS (TnI FS) and the ratios of p-AMPKα/AMPKα and p-Smad3/Smad3, while advancing the protein expressions of LC3II/LC3I and troponin I-SS (TnI SS) in the gastrocnemius tissues (Figure A1(b and c), Appendix file 1).

**Figure 4:**
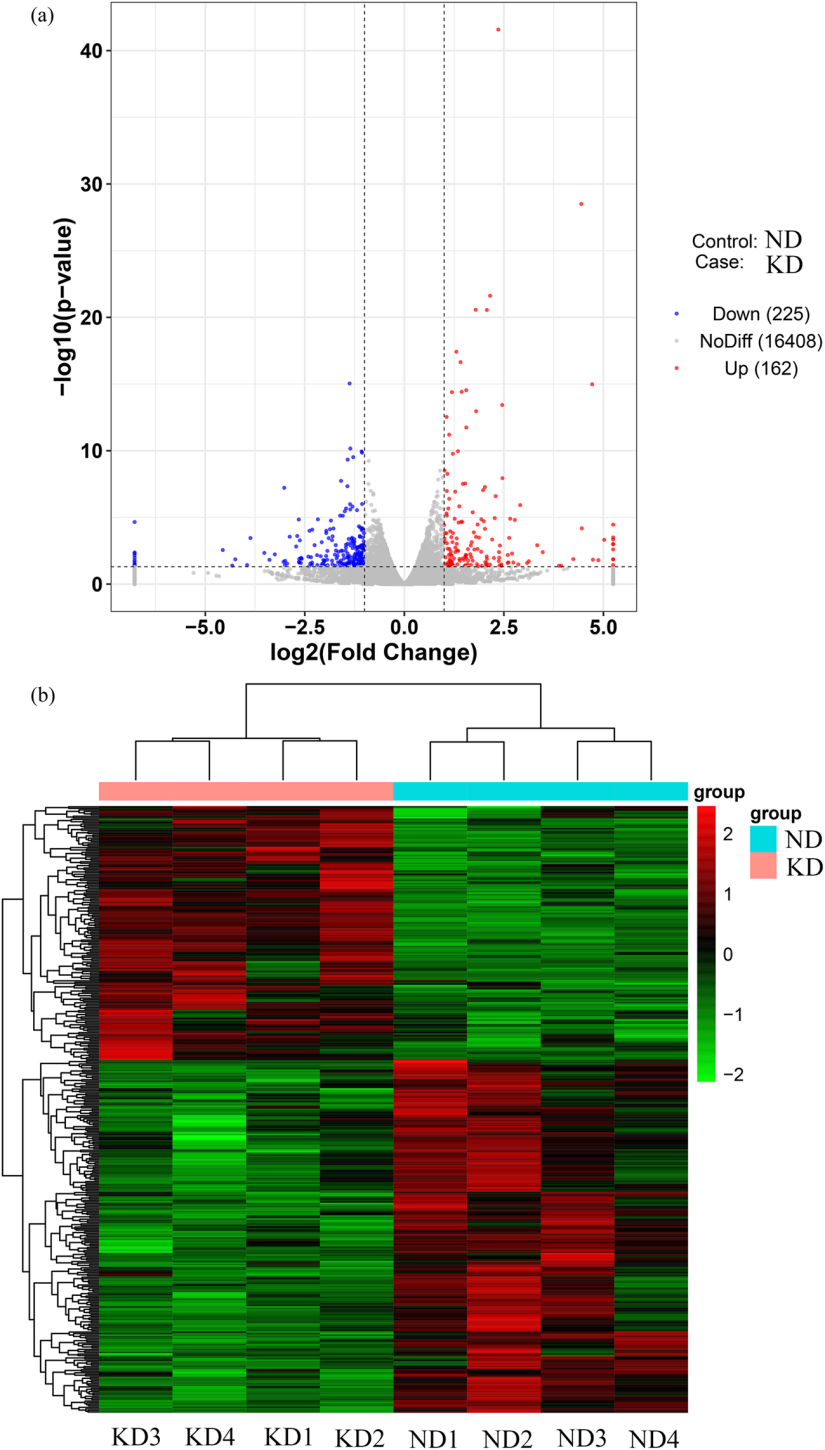
Identification of DEGs in gastrocnemius tissues in KD treated mice. (a) Volcano plot: two black vertical lines represent 1.5 times upregulation and 1.5 times downregulation, green horizontal lines indicate *P*-value of 0.05. (b) Heatmap showing the expressive pattern of DEGs. Red represents upregulated genes and green represents downregulated genes.

### 3.5 Identification and functional enrichment analysis of DEGs of gastrocnemius tissues in KD-treated mice

**Figure 5:**
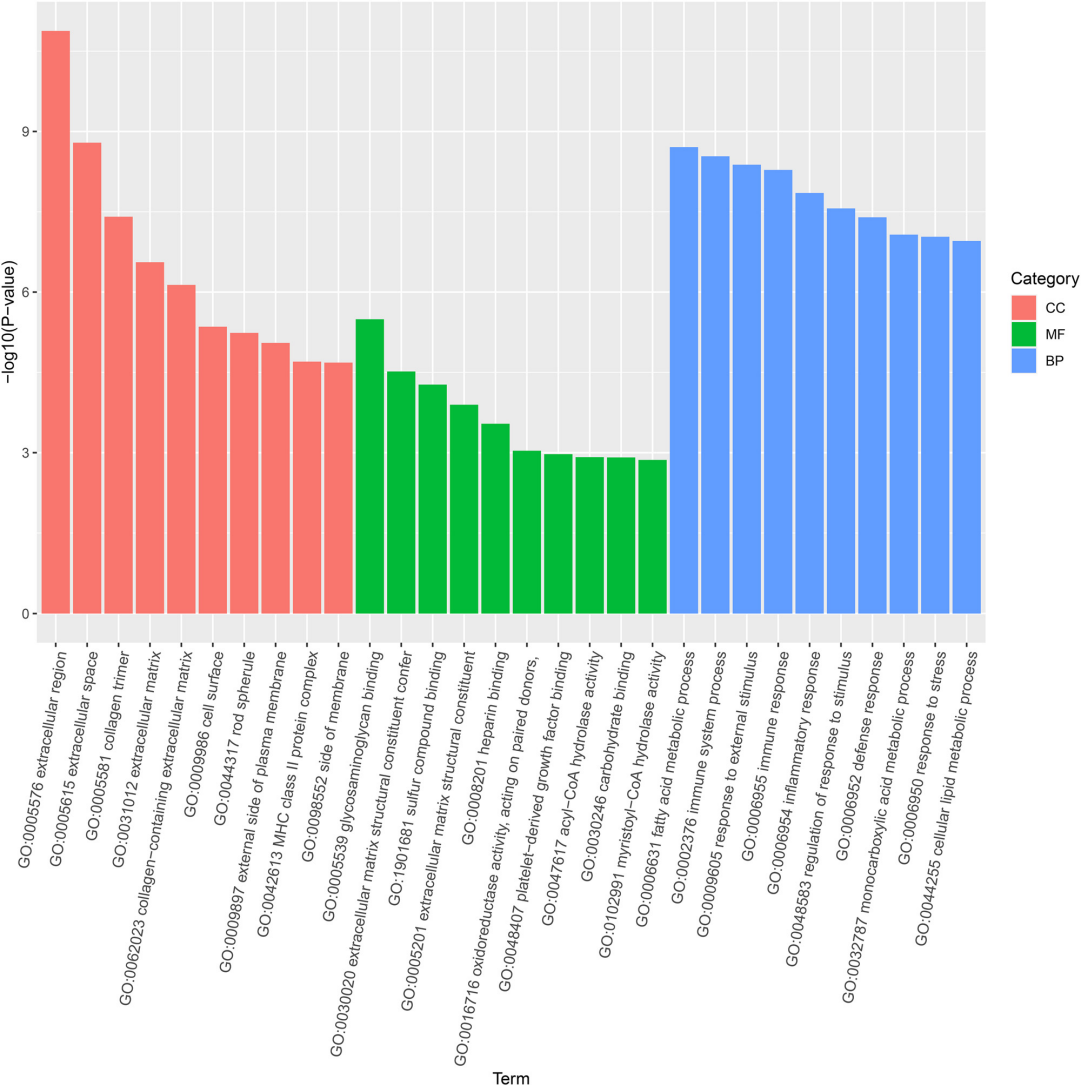
GO analysis of DEGs in mice after KD treatment.

**Figure 6:**
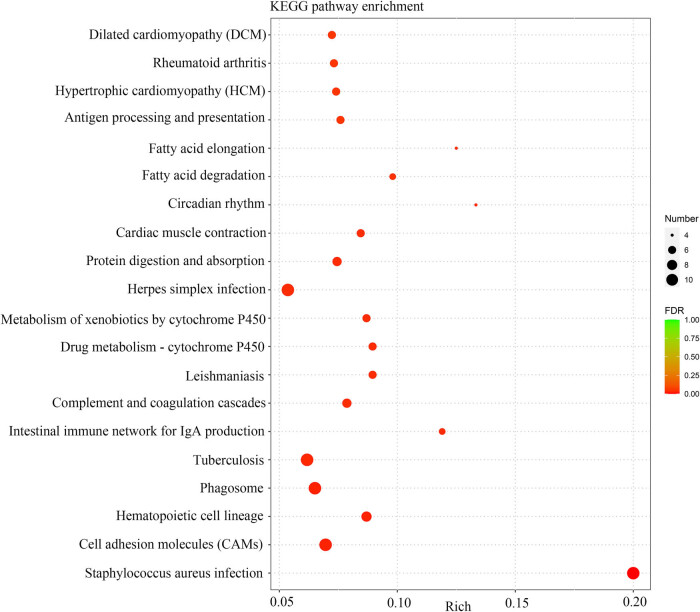
KEGG pathway analysis of DEGs in mice after KD treatment.

The volcano plots of Figure 4a exhibited the DEGs in two gastrocnemius samples. Relative to the ND group, 162 genes showed high expression and 225 genes displayed low expression in the KD group. The cluster analysis of DEGs in the results presented that the top 100 genes with the lowest *P* value were applied for mapping, among them, red represented upregulated genes and green manifested downregulated genes (Figure 4b). Afterward, GO and KEGG enrichment of DEGs was done. Biological processes, cellular components, and molecular functions presented the top ten signaling pathways, respectively (Figure 5). The KEGG enrichment analysis scatter plot was based on the enrichment significance (*P*-value) of the top 20 pathways for plotting (Figure 6).

**Figure 7:**
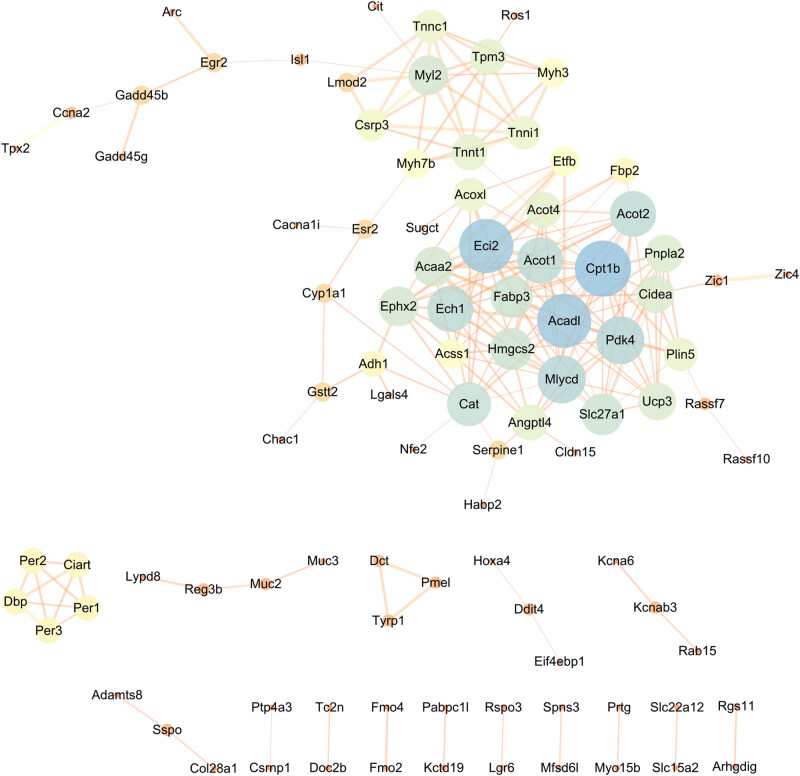
PPI network of upregulated DEGs and identification of hub genes. We mapped upregulated DEGs to the STRING database to construct a PPI network, and then re-imported these interactive network data into Cytoscape software. PPI network composed of 97 nodes and 210 edges. Hub genes obtained by the Degree algorithm, Cpt1b, Acadl, Eci2, Mlycd, and Pdk4 were considered to be key upregulated genes.

### 3.6 Identification of hub genes of upregulated and downregulated in PPI network

**Figure 8:**
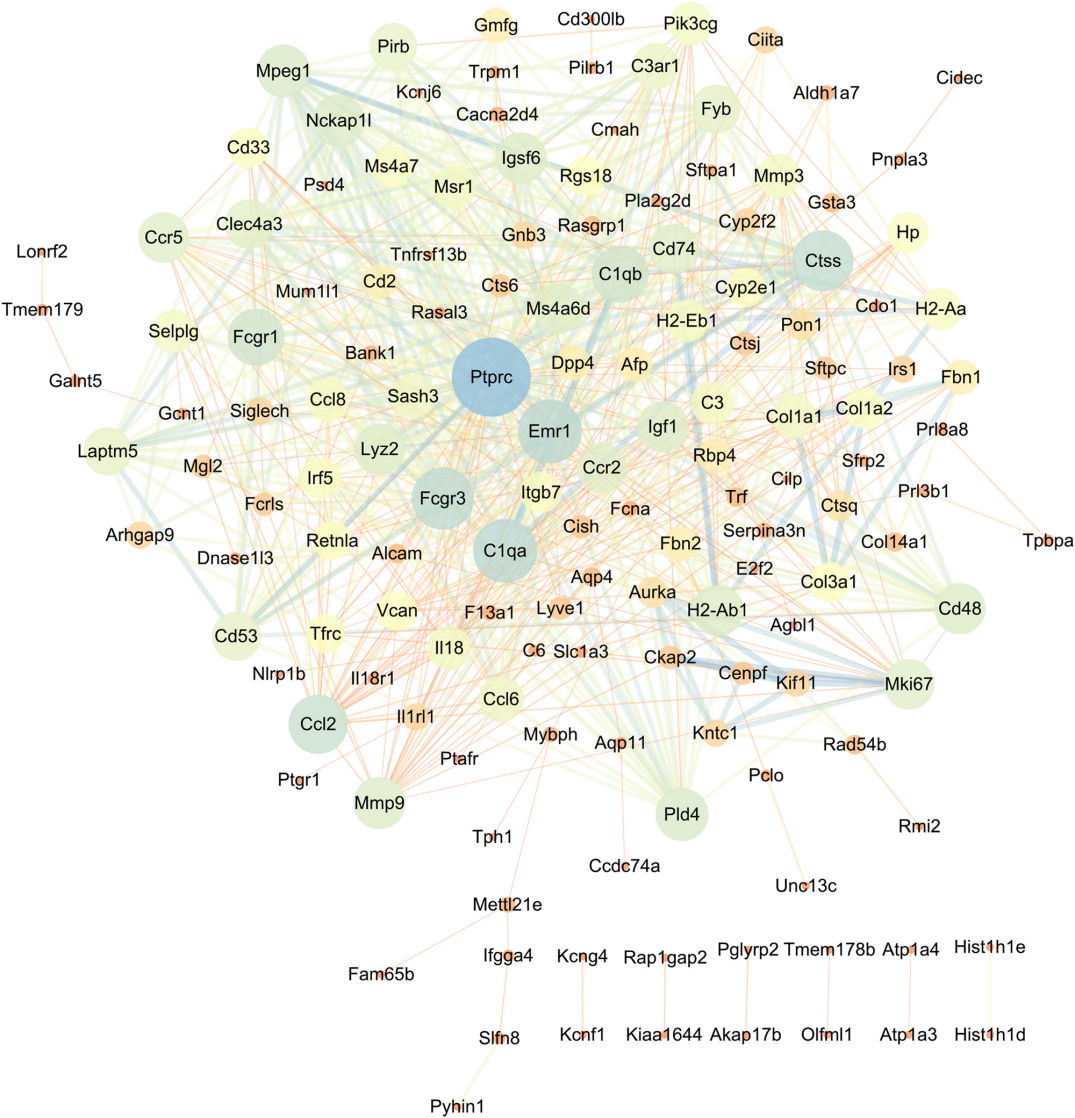
PPI network of downregulated DEGs and identification of hub genes. We also mapped downregulated DEGs to the STRING database to construct a PPI network and re-imported them into Cytoscape software. PPI network composed of 149 nodes and 684 edges. Hub genes obtained by Degree algorithm, Ptprc, C1qa, Emr1, Fcgr3, and Ctss were considered to be key downregulated genes.

We mapped upregulated DEGs to the STRING database to establish a PPI network, and then re-imported these interactive network data into Cytoscape software. The PPI network is composed of 97 nodes and 210 edges. Hub genes acquired by the Degree algorithm, carnitine palmitoyltransferase 1B (Cpt1b), long-chain acyl-CoA dehydrogenase (Acadl), enoyl-CoA-(delta) isomerase 2 (Eci2), malonyl-CoA decarboxylase (Mlycd), and pyruvate dehydrogenase kinase 4 (Pdk4) were seen as key upregulated genes (Figure 7). We also mapped the downregulated DEGs to the STRING database to build a PPI network and re-imported them into Cytoscape software. PPI network composed of 149 nodes and 684 edges, and protein tyrosine phosphatase receptor type C (Ptprc), complement C1q (C1qa), epidermal-growth-factor-like-module receptor 1 (Emr1), Fc gamma receptor III (Fcgr3), and cathepsin S (Ctss) were regarded as key downregulated genes (Figure 8).

## 4 Discussion

Mounting reports illustrated that KD could control body weight on the premise of providing sufficient energy for the body, so KD may have a certain application value in sports involving weight classes [24,25]. However, research on the relationship between KD and exercise is still very limited. In this study, C57BL/6 mice were used as the research objects. After 4 weeks of feeding, the results displayed that the weight of mice in the KD group was lower than the ND group from the second week, which was coincident with the results of other literatures [26,27]. These results illuminated that KD could lessen the body weight of mice under the condition of equal energy feeding.

KD augmented the level of blood ketone and decreased the level of Glu in mice, suggesting that the increase of ketone bodies as energy supply substances is one of the main manifestations of the KD animal model [4]. The ketone body is an intermediate product of fatty acid oxidation in the liver, and changes in the blood ketone body may have an impact on the energy metabolism of the body [28]. A relevant study pointed out that the utilization of the ketone body by extrahepatic tissues and organs in ND mice after exercise is lower than the production level of the ketone body, so blood ketone level increases after exercise [29]. Weight-bearing swimming is a considerable indicator for evaluating exercise endurance in mice [23]. The results of this experiment found that there was no notable difference in weight-bearing swimming time and distance between the KD group and ND group, which was consistent with the results reported by Helge et al. [30], and the possible reason was that a high-fat diet could change the utilization rate of metabolic substrates and increase the fat oxidation rate during exercise [31]. In addition, there was no obvious change in blood Glu level before and after weight-bearing swimming in the KD group and ND group, suggesting that KD had no evident effect on the exercise tolerance of mice, and the blood Glu level remained relatively stable before and after exercise.

We also assessed blood ketone levels in mice before and after weight-bearing swimming. In this study, the blood ketone level of mice in the ND group after swimming was effectively enhanced than those before swimming, which was in line with the increase in blood ketone level after exercise; while there was no distinct difference in the blood ketone level of mice in the KD group before and after swimming, indicating that feeding KD for 4 weeks changed the ability of mice in the KD group to utilize ketone bodies, namely “fat adaptation” [14]. In addition, plasma metabolic parameters (TC, TG, FFA, β-HB, AST, and ALT) were measured in this study to characterize the effect of KD on metabolism. It has been reported in the literature that after 9 weeks of administration of KD to mice, a significant decrease in body weight can be observed, plasma FFA and ketone body are notably increased, and Glu is evidently decreased [32]. β-HB is mainly derived from the oxidation of fatty acids and exported to the surrounding tissues for energy [4]. The concentration of β-HB was largely enhanced in the KD group, indicating the utilization of fat or ketone body [33,34]. By conducting ELISA analysis, the results illustrated that the contents of serum TC, FFA, and β-HB were higher and the contents of serum TG and AST were lower in the KD group when compared to the ND group. These results unveiled that KD transferred energy substrates from Glu to fat.

The literature indicated that the changes in the expression levels of different functional signaling pathways, such as AMPKα, Smad3, and PGC-1α, have the function of regulating the composition of skeletal muscle fiber types [35]. PGC-1α is a key factor in promoting mitochondrial biosynthesis [36]. AMPK is a key sensor of skeletal muscle energy status and modulates Glu and fatty acid metabolism [37]. AMPK can regulate PGC-1α through phosphorylation and deacetylation, and participate in mitochondrial function and biosynthesis [38,39]. High-fat diet feeding has been manifested to weaken the expression and activity of PGC-1 and AMPK as well as downstream signaling in skeletal muscle [40]. The results by western blot illustrated that KD was found to alleviate the protein level of PGC-1α and the ratios of p-AMPKα/AMPKα and p-Smad3/Smad3 in the gastrocnemius tissues. In order to further verify the effect of KD on muscle fiber types, this experiment detected TnI SS, which can reflect the content of slow-twitch muscle fibers in skeletal muscle, and TnI FS, which can reflect the content of fast-twitch muscle fibers in skeletal muscle. The contents of TnI SS and TnI FS in different types of muscle fibers were detected by western blot, and it was found that the expression of TnI SS was increased and the expression of TnI FS was decreased in the KD group in the gastrocnemius, indicating that KD can effectively increase the expression of slow muscle fibers in mixed-type muscle fibers.

In addition, autophagy is a universal life phenomenon of eukaryotic cells, responsible for the removal of damaged and dysfunctional organelles. Autophagy pathway activation is characterized by the formation of autophagosomes in LC3-containing cells. P62 is a selective substrate of LC3 and is continuously consumed during autophagic activation. The level of expression of p62 and LC 3 indicators can be used to assess the degree of autophagy pathway activation [41]. Mu et al. found that KD intervention could mitigate pulmonary fibrosis *in vivo* via upregulating the expression of LC3 II/LC3 I and Beclin1 and downregulating the expression of P62 [42]. This research found that KD effectively repressed the protein level of P62, while advancing the ratio of LC3II/LC3I in the gastrocnemius tissues, illuminating that KD triggered autophagy in C57BL/6 mice.

With the development of RNA-seq technology, second-generation sequencing has been broadly utilized in genome research [43]. To further analyze the effect of KD on genomic changes in C57BL/6 mice, RNA-seq analysis was performed on gastrocnemius samples from mice in the KD and ND groups. As a result, a total of 387 DEGs were filtered between ND and KD-treated groups. Importantly, the GO and KEGG pathway enrichment analysis found that DEGs participated in fatty acid elongation, fatty acid degradation, fatty acid metabolic process, etc. It is well known that fatty acids are closely related to skeletal muscle strength and exercise endurance [44,45]. In future research, this study will explore the effects of KD on skeletal muscle strength and exercise endurance through fatty acid metabolism-related pathways. Additionally, hub genes were obtained by using Cytoscape software, Cpt1b, Acadl, Eci2, Mlycd, Pdk4, Ptprc, C1qa, Emr1, Fcgr3, and Ctss were considered to be key genes.

By searching the literature related to skeletal muscle strength and exercise endurance of the above ten hub genes, Cpt1b, Acadl, Eci2, Mlycd, Pdk4, Ptprc, C1qa, Emr1, Fcgr3, and Ctss are all closely correlated with skeletal muscle strength and athletic endurance. Cpt1b is a mitochondrial enzyme responsible for the formation of acylcarnitine, which helps to regulate fatty acids β-oxidation [46,47]. It was reported that Cpt1b is highly expressed in skeletal muscle, heart, and adipose tissues [48]. Acadl is a key enzyme for mitochondrial fatty acid oxidation, which is notably lower by exercise in Nmrk2^−/−^ mice [49,50]. Eci2 is a peroxisomal and/or mitochondrial protein and Eci2 knockdown lessens Glu consumption [51,52]. Mlycd is a considerable enzyme for fatty acid metabolism and acyl-CoA synthesis and metformin restrains lipid accumulation in obese adults via the upregulation of Mlycd [53]. Pdk4 is a crucial regulator of cellular energetic metabolism, which is widely expressed in skeletal muscle tissues including humans and rodents [54]. Ptprc (also known as CD45) is an essential transmembrane glycoprotein on the cell surface of the hematology and immune system [55]. It was reported that exogenous C1qa weakened skeletal muscle regeneration in young mice, while C1qa destruction restored aging-related muscle regeneration injury [56]. Emr1, the adipocyte gene, is related to advanced age and diet-induced obesity [57]. Fcgr is a protein family expressed by a variety of immune cells, among which Fcgr3 is one of the members of the Fcgr protein family [58]. Ctss is a key member of the cysteine protease family, and its inhibitor could reduce fat production and liver fat accumulation in obese mice mediated by a high-fat diet [59]. The expression and activity of Ctss were upregulated in the skeletal muscles of mice with Duchenne muscular dystrophy [60]. Therefore, their correlations with skeletal muscle strength and exercise endurance warrant further investigation, as these genes are potential targets of KD.

## 5 Conclusions

This study provides a reference for the dietary pattern of KD to lose weight, and maintain muscle strength and exercise capacity, and its mechanism is related to the regulation of skeletal muscle fiber type-related proteins and autophagy-related proteins. However, whether longer-term KD affects aspects such as skeletal muscle strength remains to be further investigated.
